# pH-dependent defense responses in eggplant: growth performance and resistance to *Verticillium dahliae*

**DOI:** 10.3389/fpls.2026.1785225

**Published:** 2026-03-12

**Authors:** Muhammad Awais, Tianqi Wang, Jitong Xu, Xin Bu, Xing Li, Chaoge Yu, Muhammad Sohail Khan, Hongdan Fu, Zhouping Sun

**Affiliations:** 1 College of Horticulture, Shenyang Agricultural University, Shenyang, China; 2 Key Laboratory of Protected Horticulture of the Education Ministry, Shenyang, China; 3 Department of Horticulture, Faculty of Agriculture, Gomal University, Dera Ismail Khan, Khyber Pakhtunkhwa, Pakistan

**Keywords:** eggplant, *Verticillium dahliae*, antioxidase, *SmeGLU*, SmeGPX

## Abstract

Eggplant is a globally important vegetable crop, yet its intensive greenhouse cultivation often leads to soil acidification, which exacerbates the incidence of Verticillium wilt. *Verticillium dahliae* (*V. dahliae*) is a major soil-borne pathogen that severely reduces eggplant growth, however, the defense responses of eggplant under different pH conditions and the mechanistic basis underlying pH-mediated resistance are still untouched and unclear. Therefore, the physiological and molecular responses of eggplant to *V. dahliae* inoculation under two soil pH conditions (5.5 and 6.5 VD/CK) was investigated. Soil pH profoundly influences disease severity and plant defense activation. *V. dahliae* inoculation at pH 5.5 led to a high disease index of 59.67%, significantly stunting plant growth. In contrast, a near-neutral pH of 6.5 promoted robust plant growth even under pathogen challenge. Defense responses were potentiated at pH 6.5, as evidenced by a significant increase in the activity of key defense enzymes (CAT, PAL, POD, PPO, SOD, β-1, 3-glucanases) and the upregulation of defense-related genes *SmeGLU* and *SmeGPX*. Subcellular localization confirmed that this enhanced defense correlated with a stronger nuclear accumulation of defense proteins in inoculated plants at pH 6.5. pH modulates resistance not only by reducing disease severity but also by enhancing transient protein expression and subcellular defense responses. Results demonstrated that a near-neutral soil pH of 6.5 is optimal for priming the eggplant defense system against *V. dahliae*. These findings reveal a critical link between soil pH and innate immunity in eggplant, offering new avenues for leveraging pH management and defense-related genes for crop improvement.

## 1 Introduction

Eggplant (*Solanum melongena* L.) also known as brinjal is a widely cultivated and globally significant vegetable crop. Botanically, eggplant is a fruit but used as a vegetable, with its intensive cultivation increasingly conducted within protected systems (Zhou et al., 2023). China, the dominant producer, accounts for approximately 65% of the world’s output (Al Salmi et al., 2020). However, the sustainability of this intensive cultivation is threatened by soil acidification. A pervasive abiotic stressor driven predominantly by different factors including acid rain, continues cropping, and the overuse of synthetic fertilizers especially the nitrogenous ones (Wang et al., 2022). Acidification detrimentally alters soil chemistry, impairs nutrient availability, and disrupts root architecture, ultimately compromising plant growth, yield, and quality (Wang et al., 2025).

Beyond its direct impact on plant physiology, soil pH is a crucial determinant of plant-fungal pathogen interactions (Li et al., 2017; Wang et al., 2024). Optimal pH supports plant health and nutrient absorption, even slight deviations can shift the outcome of infection by modulating both fungal virulence and host immunity (Wang et al., 2024; Zhu et al., 2021). These pH-mediated shifts in host-pathogen dynamics are particularly evident in soil-borne vascular diseases e.g. *V. dahliae*, where changes in the rhizosphere environment directly influence pathogen survival, infection efficiency, and host defense responses (Fang et al., 2012; Rampersad, 2010). *V. dahliae*, which ranks among the most destructive diseases of Solanaceous crops like eggplant and causes verticillium wilt (Zhu et al., 2021). *V. dahliae* colonizes the xylem, leading to wilting, stunting, and yield losses exceeding 60% (López-Moral et al., 2021). *V. dahliae* isolates from artichoke and pumpkin reportedly more severe and causes huge yield losses in acidic conditions around pH 5.0 (Kabir et al., 2004; Rampersad, 2010) compared to higher pH conditions where disease severity was lower with higher disease resistance in plants (Fang et al., 2011). Similarly, potato scab was controlled by maintaining soil pH around 5.5 to 6.5, so it should be suitable for plant not for the diseases (Shimono, 1990). Furthermore, an opposite correlation was observed between soil pH and disease severity in strawberry fields in Western Australia (Fang et al., 2011), with pH around 6.8 was the optimal condition against the fungal pathogen *Fusarium* disease resistance (Fang et al., 2012). *V. dahliae* is notoriously difficult to control due to wide host range and high genetic diversity that drives adaptation to wide pH ranges (Li et al., 2024a; Wang et al., 2024).

The soil pH is a critical environmental modulator of plant innate defense, often determining disease outcomes by shaping both microbial activity and host resistance capacity. Field observations revealed a complex, non-linear relationship, where an optimal pH range often between 5.5 and 6.8 peaks plant immunity against various pathogens like potato scab and *Fusarium* spp (Fang et al., 2012, Fang et al., 2011). A primary mechanism underlying pH-dependent susceptibility is the direct modulation of key defense-related enzymes against pathogens causing diseases. These include antioxidant and defense enzymes, such as peroxidase (POD) and polyphenol oxidase (PPO), which are known to enhance cell structure and contribute to defense by forming lignin and phenolic compounds as barriers against pathogens (Nasr-Esfahani et al., 2020). Together, catalase (CAT), POD, and superoxide dismutase (SOD) are key components of the plant’s enzymatic system, which plays a crucial role in defending against pathogen-induced oxidative stress (Monazzah et al., 2018; Zhang L. et al., 2015; Zhou et al., 2012). Recent studies of plant-pathogen interactions in pepper, cucumber, pumpkin, and tomato have documented a significant role for enzymes in defense responses (Mohammadbagheri et al., 2021; Najafi et al., 2024; Nasr-Esfahani et al., 2020). Crucially, suboptimal soil pH can impair these defenses by downregulating key biochemical enzymes such as POD, CAT, chitinase, β-1,3-glucanase, and Superoxide dismutase (SOD), as demonstrated in rice and cucumber (Shi et al., 2006; Zhang et al., 2015). Maintaining an optimal rhizosphere pH may therefore be essential for preserving this biochemical defense layer and enhancing the plant’s innate resistance mechanisms (Bidzinski et al., 2016; Singh et al., 2020).

The expression of plant defense genes is sensitive to environmental pH (Lemaître et al., 2008; Singh et al., 2020) as it can directly modulate the transcriptional programs governing plant defense. For instance, in *Arabidopsis thaliana*, the expression of genes such as *TOUCH4, GATA2*, and *EXPANSIN A17* is regulated by pH variation (Lager et al., 2010). These mechanisms are conserved in crops, where resistance to pathogens like *V. dahliae* involves the deployment of specific genetic components. In cotton and tomato, this involves the upregulation of genes for metabolic reorganization (*GhGDH2*) or pathogen recognition (*V1, V2, Ve*), the latter promoting lignin accumulation to restrict pathogen survival (Fradin et al., 2009; Xiong et al., 2021). A central component of this defense arsenal in plants like eggplant is the induction of pathogenesis-related (PR) proteins, particularly β-1, 3-glucanase (GLU, PR-2) and chitinases (PR-3, PR-4, PR-8, PR-11), which have direct antifungal activity (Mahesh et al., 2017; Zhou et al., 2012). Beyond pathogen suppression, they contribute to cell wall fortification, a critical defensive barrier. For example, in wheat, POD and β-1, 3-glucanase act synergistically to enhance lignification and strengthen the cell wall against infestation (Zondo and Mafa, 2025).

While the individual effects of pH on plant growth and yield are documented, the specific interplay between eggplant-*V. dahliae* interactions and the induction of eggplant’s defense mechanism is entirely uncharacterized under soil pH conditions. It is unknown how a gradient of soil pH influences the transcriptional regulation of key defense genes and the subsequent biochemical responses in this pathosystem. This study is a follow-up to our group’s previous research, which screened seven (4.5, 5.0, 5.5, 6.0, 6.5, 7.0 and 7.5) pH conditions *in vitro* and in the greenhouse to identify disease-suppressive and disease-supportive levels. Based on the disease index and suppressiveness results from that work, we selected two pH levels (5.5 and 6.5, with Control (CK)) to study the underlying enzymatic and gene expression patterns, along with transient protein concentrations. Therefore, the aim of this study is to elucidate the physiological and molecular mechanisms underpinning the pH-dependent resistance of eggplant to *V. dahliae*. We hypothesize that an optimal soil pH will prime eggplant’s defense by synchronously enhancing growth, activating a robust enzymatic system, and upregulating resistance genes and protein. By integrating analyses of plant performance, we seek to provide a holistic understanding of how soil management can be leveraged to bolster innate plant immunity.

## 2 Materials and methods

### 2.1 Experimental material and design

The eggplant cultivar ‘Xian Green’ and *Verticillium dahliae* were used as experimental materials. The pathogen was isolated from a diseased eggplant and identified as *V. dahliae* isolate Le1333. Growth characteristics across seven (7) pH-adjusted potato dextrose agar (PDA) and potato dextrose broth (PDB) media, as well as pathogenicity under seven simulated soil pH conditions (4.5–7.5), were also studied, where pH 5.5 shows disease supportive and 6.5 disease suppressive conditions. Based on these results, eggplants were grown in soils with pH 5.5 and 6.5, with and without *V. dahliae* inoculation, to evaluate plant growth responses, gene expression patterns, and to perform subsequent western blotting using tobacco plants under the same conditions.

Soil was sterilized and inoculated using a cornmeal-sand mixture (CMS) containing *V. dahliae* microsclerotia (MS) at a density of 3 × 10^7^ MS g-¹ (Anguita-Maeso et al., 2021). Each pot was filled with 1.25 kg of sterilized soil and 25 g of the inoculated CMS mixture. Control (CK) pots received the same mixture without the pathogen. Uniform, healthy four-leaf-stage eggplant seedlings were surface-sterilized with 1% sodium hypochlorite, rinsed thoroughly, and transplanted into the pots. To adjust the soil pH to 5.5 and 6.5, different concentrations of H_2_SO_4_ (0.70 and 0.20 ml/L) solutions were used. To ensure the stability of the soil pH over the 60-day experimental period, the pH of both the inoculated and CK treatments was measured every 7 days. Additional H_2_SO_4_ solutions were added as needed to maintain the targeted pH levels (Wang et al., 2025; Zhang et al., 2022). The pH of the neutral soil was 7.23. The initial, middle and final pH values for both treatments during the experiment are provided in **Supplementary Table 1**.

### 2.2 Measurement and analytical procedures

The plants were transplanted in *V. dahliae* inoculated (VD) and non-inoculated control (CK) soil and maintained in the greenhouse for 60 days. After 60 days of inoculation (dpi), Plant growth indices like, plant height, total plant biomass (g), and root biomass were determined and root volume were assessed using a root scanner (WinRHIZO™ 5.0 STD 4800, Canada). Root samples for gene expression analysis were flash-frozen in liquid nitrogen and later stored in -80 °C refrigerator for subsequent enzymatic activities and RNA extraction.

#### 2.2.1 Disease index (DI%) of eggplant

Disease progression was monitored over a five-week period, commencing 30 dpi and concluding at 60 dpi. At seven-day intervals, DI and severity were evaluated by visually assessing the percentage of foliar disease symptoms. A standardized 0–4 rating scale was employed, where; grade 0 represented No symptoms, grade 1 (≤25% leaf yellowing), grade 2 (50% leaf yellowing with marginal shrivelling), grade 3 (>50% leaf wilting and shrivelling), and grade 4 complete foliar collapse or plant death (Ogundeji et al., 2022).

The given equation was employed for calculating DI %.


$$\text{DI}=\sum \left[\frac{\left(\text{No}.\text{ of diseased plants in each grade}\times \text{Corresponding disease grade}\right)}{\left(\text{Total No}.\text{ of plants}\times \text{Highest disease grade}\right)}\right]\times 100$$

#### 2.2.2 Determination of enzymatic activities

0.5 g root samples from CK and *V. dahliae* inoculated plants were ground in a liquid Nitrogen followed by homogenizing in the mixture of 0.1 mmol.l^-1^ potassium phosphate buffer (pH 7.5), EDTA 1 mmol.l^-1^, PMSF 2 mmol.l^-1^, and Triton 0.1%, and 1% polyvinyl polypyrrolidone (w/v). The mixture was then centrifuged at 12298 g for 10 min at 4 °C and the supernatant was stored at -20 °C. The supernatants were collected and used to measure total protein content and enzymatic activity (Najafi et al., 2024).

Total protein content was quantified using the Bradford method (1976) with bovine serum albumin (BSA) as the standard (**Supplementary Figure 1**). POD activity was determined following Najafi et al. (2024), measuring absorbance at 420 nm. CAT activity was assayed as per Monazzah et al. (2018) in a reaction mixture containing 50 mM potassium phosphate buffer (pH 7.0), 15 mM H_2_O_2_, and 20 µL root extract, with activity recorded at 240 nm as µmol H_2_O_2_ decomposed min-¹ mg-¹ protein. Polyphenol oxidase (PPO) activity was measured according to Mohammadbagheri et al. (2021) using catechol (20 mM) in sodium phosphate buffer (pH 6.8), with absorbance monitored at 430 nm and expressed as µmol catechol oxidized min-¹ mg-¹ protein.

Phenylalanine ammonia lyase (PAL) activity was evaluated following Mohammadbagheri et al. (2021) by incubating the reaction mixture (containing L-phenylalanine and root extract) at 37 °C for 60 m. The reaction was terminated with 6 M HCl, and cinnamic acid production was measured at 290 nm as µmol min-¹ mg-¹ protein. SOD activity was assayed using riboflavin-NBT in phosphate buffer (pH 7.8) under light exposure, with absorbance read at 560 nm and expressed as units mg-x207B;¹ protein.

β-1, 3-glucanase activity was determined via Najafi et al. (2024) using laminarin as substrate, with glucose release quantified at 500 nm using DNS and reported as µmol glucose min-¹ mg-¹ protein.

#### 2.2.3 Determination of defense metabolites

Total phenolic (TP) content was estimated via the Folin-Ciocalteu method, with absorbance measured at 725 nm and expressed as µg gallic acid equivalents g-¹ FW. Lignin content was analyzed following through sulfuric acid digestion, with gravimetric quantification after oven drying.

#### 2.2.4 Determination of oxidative damage and membrane integrity

Malondialdehyde (MDA) Content was measured through thiobarbituric acid (TBA) method (Zhou et al., 2012). The optical density of supernatants was scored at 450, 532, and 600 nm. Blank controls were prepared without crude enzymes.

Membrane permeability was assessed following the methodology described by Wu et al. (2022), and was calculated using the following equation:


$$EL=\frac{\text{EC}1}{\text{EC}2}\times 100$$

Where: EC1 = initial electrical conductivity (EC) measured after soaking at 25 °C for 30 min.

EC2 = final EC measured after hot water bath treatment for 30 min.

#### 2.2.5 Primer designing and RT-qPCR analysis

We selected defense genes spanning immune signaling, mitogen-activated protein kinases (MPK1), auxin/indole-3-acetic acid (IAA27), antioxidant activity, glutathione peroxidases (GPX), direct antifungal action, chitinase (CHT), β-1, 3-glucanase (GLU), pathogenesis related protein 1 and 5 (PR1, PR5), and hormone biosynthesis lipoxygenase (LOX) to determine how contrasting soil pH levels (5.5 vs 6.5) modulate the coordination of eggplant’s immune response to *V. dahliae.* Candidate genes were identified based on a comprehensive review of eggplant disease-resistance literature (Ali et al., 2022; El-Habashy et al., 2024; Mahesh et al., 2017; Pang et al., 2021; Zhang et al., 2025). Corresponding gene sequences were extracted from the Eggplant Genome Database and NCBI, and primers were designed using Primer3 (**Supplementary Table 2**).

Total RNA was extracted from 0.5 g of eggplant root tissues using the Ultrapure RNA Kit (CWBIO, Beijing, China) and reverse-transcribed with the SuperRT III All-in-One RT Mix with gDNA Remover (Biosharp, Hefei, China), following the manufacturers’ protocols. RNA and cDNA quality and concentration were assessed using a NanoDrop spectrophotometer.

RT-qPCR was performed with three biological replicates per treatment using 2× Universal SYBR Green Fast qPCR Mix (ABclonal, Wuhan, China). Each 20 μL reaction contained 10 μL SYBR Green mix, 7 μL RNase-free water, 1 μL cDNA, and 1 μL of each primer. Actin was used as the internal reference. Relative expression levels were calculated using the 2^-ΔΔCT^ method (Livak and Schmittgen, 2001).

#### 2.2.6 Subcellular localization

For subcellular localization, the putative promoter regions and the coding sequences of *SmeGPX* and *SmeGLU* were amplified. These fragments were cloned into a promoterless GFP-expression vector using an In-Fusion cloning kit, generating transcriptional fusions where GFP expression is driven by the native *GPX* and *GLU* promoters. These constructs were introduced into *Agrobacterium tumefaciens* GV3101 and infiltrated into tobacco leaves for transient expression, planted in 5.5 and 6.5 pH with and without *V. dahliae* inoculation as a spore suspension (1× 10^6^). Plants were kept in the dark for 24 h, after infiltration, before being returned to normal growth conditions. Leaf samples were collected at 4 dpi and analyzed using a ZEISS LSM 780 laser scanning microscope following the protocols by Zhang et al. (2023).

#### 2.2.7 Western blot analysis

Transiently transformed tobacco plants were transplanted in 5.5 & 6.5 pH soil for 4 days and protein were isolated from tobacco leaves following the methods proposed by Wang et al. (2019). A total of 40 μL protein was mixed with 5×SDS loading buffer (CEBIO, China) and denatured at 95 °C for 5 min and then separated on 4 to 20% gel (FuturePAGE™ 4-20%, ACE, China) for 40 min at 150V followed by the primary antibody (Anti-GFP tag Monoclonal antibody, Solarbio, and Anti-Actin mouse mAb antibody for reference Actin protein, Biopmk, Wuhan, China), and secondary anti-Mouse antibody (Goat Anti-Mouse IgG/HRP). Subsequent steps for immunoblotting were performed according to the methods described by Na et al. (2016).

### 2.3 Statistical analysis

Statistical comparisons were performed through one-way analysis of variance (ANOVA) followed by Tukey’s Honest Significant Difference (HSD) test at a significance level of *P* ≤ 0.05 using Origin 2021 (OriginLab, New York, USA). A detailed comprehensive principal component analysis (PCA) was performed using Origin 2021. Results are expressed as mean ± SE.

## 3 Results

### 3.1 Effect of soil acidification and *V. dahliae* inoculation on eggplant growth and disease severity

**Figure 1:**
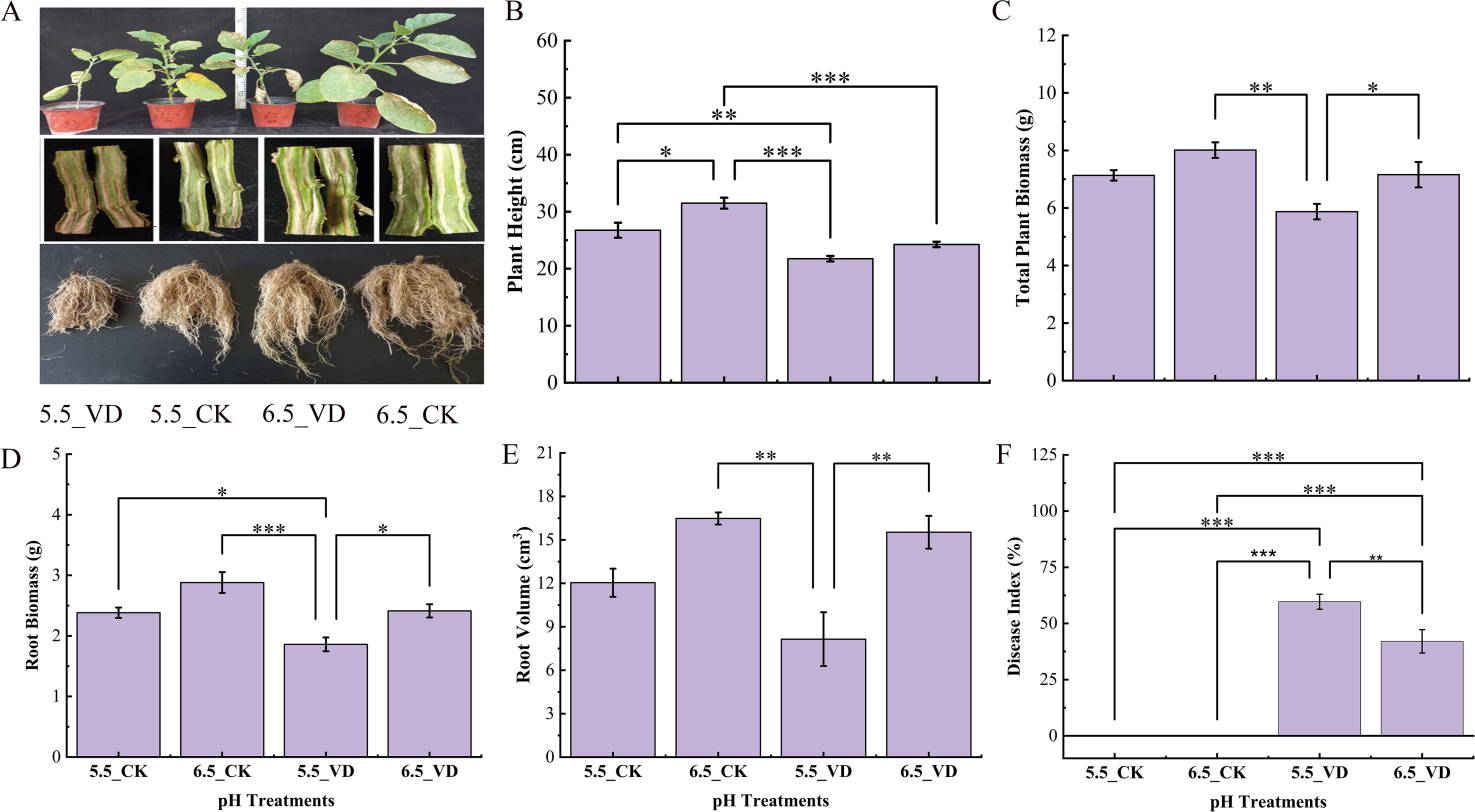
Effect of soil acidification and *V. dahliae* inoculation on plant growth of eggplant. Plant growth, longitudinally cut plant stems for *V. dahliae* disease severity, and Eggplant root growth **(A)**, Plant height **(B)**, Total plant biomass **(C)**, Root biomass **(D)**, Root volume **(E)**, and Disease index % **(F)**. Control plants (CK), *V. dahliae* inoculated (VD). Error bars indicate the mean ± standard error of the (n = 4, ***P <0.001, **P < 0.01, *P < 0.05).

Effect of soil acidification along with and without disease inoculation on eggplant plant growth was statistically significant. Above and below-ground plant growth parameters increased with rising pH levels, but growth was restricted under the dual stress of disease inoculation and low pH (**Figure 1A**). The combined stress at pH 5.5_VD synergistically reduced eggplant plant height by 31% shorter compared to the 6.5_CK plants (**Figure 1B**). Similarly, total plant biomass in the 5.5_VD treatment was 26.7% lower than the 6.5_CK, while root biomass showing a 35.4% decrease in 5.5_VD. Acidification alone (5.5_CK) also significantly suppressed growth compared to the optimal 6.5_CK condition. Furthermore, the root volume exhibited significant variations with highest values were recorded in single stressed 6.5_CK pH plants followed by 6.5_VD plants (**Figure 1E**).

Significant differences (*P* ≤ 0.05) were observed between the two pH treatments in *V. dahliae* inoculation (**Figure 1F**), whereas CK plants remained healthy with no signs of disease symptoms. The highest DI 59.67% was recorded in plants grown at pH 5.5, while the lowest (42.05%) was recorded at pH 6.5. Visual assessments of plant growth, longitudinal cut stem sections, and root development (**Figure 1A**) further demonstrated that higher soil pH (6.5) not only encouraged better plant growth but also enhance resistance to *V. dahliae*, resulting in significantly reduced disease severity, lower vascular discoloration, compared to lower pH (5.5) conditions.

### 3.2 Effect of soil acidification on eggplant defense enzymes and metabolite accumulation

**Figure 2:**
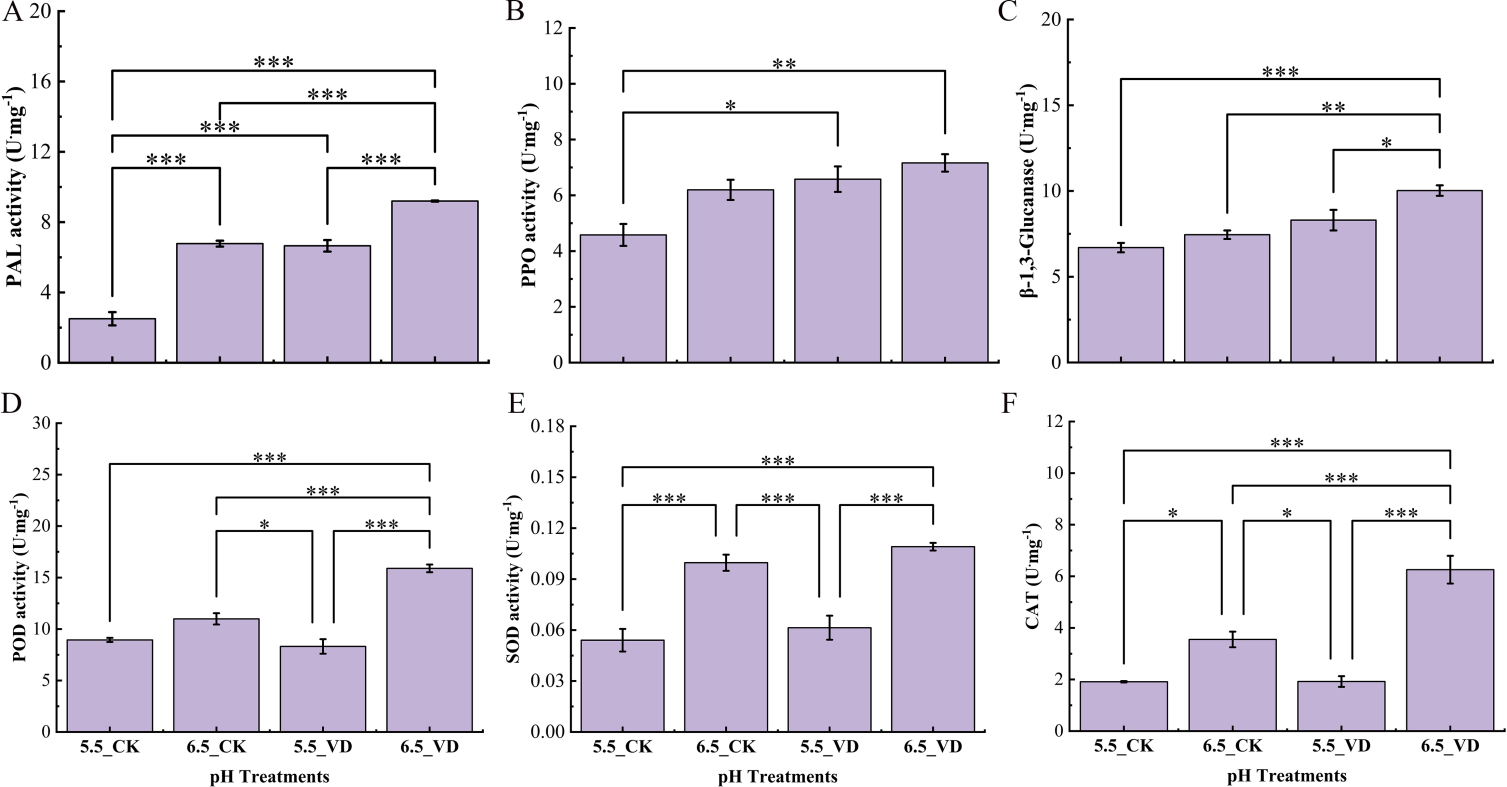
Effect of soil acidification and *V. dahliae* inoculation on eggplant enzymatic activities of PAL **(A)**, PPO **(B)**, β-1, 3-Glucanase **(C)**, POD **(D)**, SOD **(E)**, and CAT **(F)**. Control plants (CK), *V. dahliae* inoculated (VD). Error bars indicate the mean ± standard error (n = 4, ***P <0.001, **P < 0.01, *P < 0.05).

Soil acidification and *V. dahliae* inoculation had a significant (*P<* 0.05) impact on the activity of antioxidant and defense-related enzymes including CAT, PAL, POD, PPO, SOD, and β-1, 3-glucanase (**Figure 2**). The activities of these enzymes were higher in the roots of *V. dahliae*-inoculated plants at both pH 5.5_VD and 6.5_VD compared to CK at their respective pH levels. Specifically, when compared to plants at pH 5.5_CK, the plants at pH 6.5_VD exhibited increases in CAT, PAL, POD, PPO, SOD, and β-1, 3-glucanase activities of 227.1%, 267.6%, 81.6%, 56.3%, 101.8%, and 49.5%, respectively.

**Figure 3:**
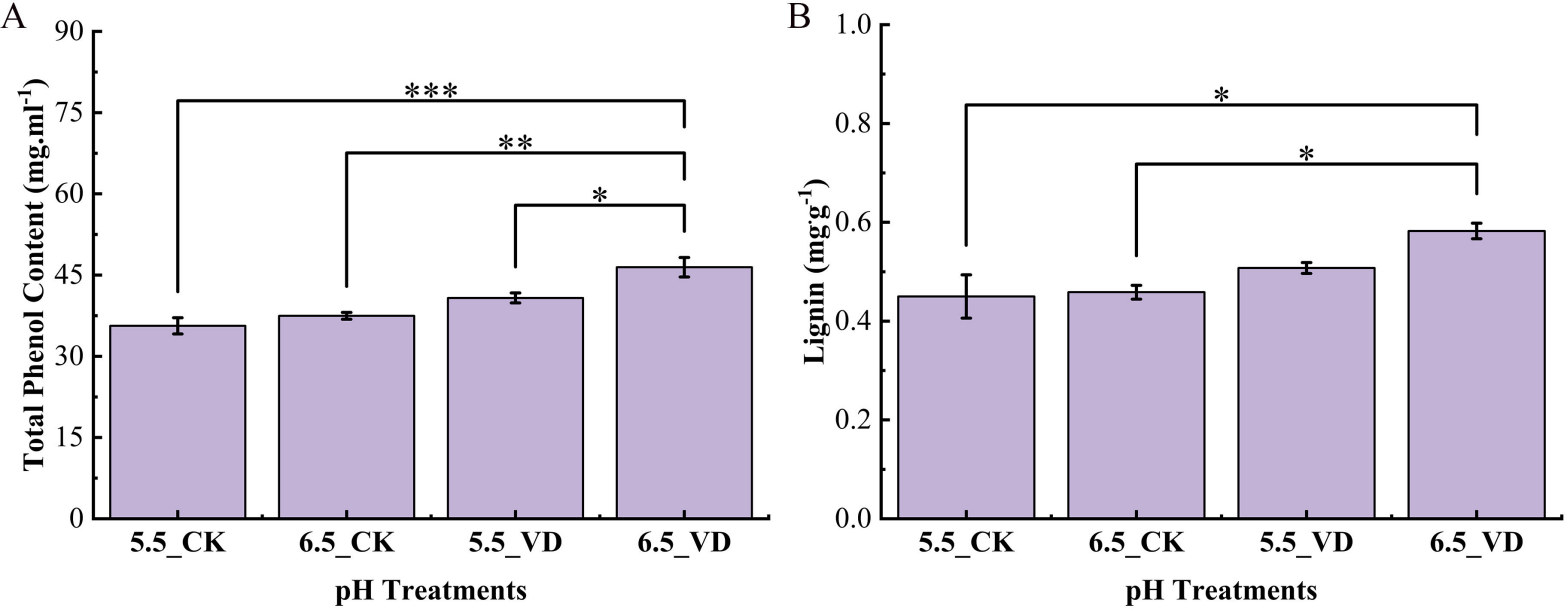
Effect of soil acidification and *V. dahliae* inoculation on total phenol content **(A)** and lignin **(B)**. Control plants (CK), *V. dahliae* inoculated (VD). Error bars indicate the mean ± standard error (n = 4, ***P <0.001, **P < 0.01, *P < 0.05).

Total phenolic (TP) content was influenced by soil pH and disease inoculation (**Figure 3A**). The highest phenolic content was induced in inoculated plants at pH 6.5_VD with 1.30-fold increases compared to 5.5_CK plants. Similarly, lignin content was also increased with higher values observed in plants at pH 6.5_VD soil (**Figure 3B**). While no significant differences were found between the CK groups. Lignin deposition was 32.1% higher in plants at pH 6.5_VD than the plants at pH 5.5_CK suggesting its accumulation in the vascular system acts as a physical barrier to limit pathogen spread.

### 3.3 Effect of soil acidification and *V. dahliae* inoculation on key disease resistant gene expression of eggplant

To assess the impact of soil pH and *V. dahliae* inoculation on eggplant defense responses, the expression of key pathogen resistance-related genes was analyzed using RT-qPCR. Among the eight genes selected for this study, *β-1, 3-Glucanase* (*GLU*) and *Glutathione Peroxidase* (*GPX*) showed significant differential expression and are presented here, however the results of remaining 6 non-significant genes are presented in **Supplementary Figure 2**.

**Figure 4:**
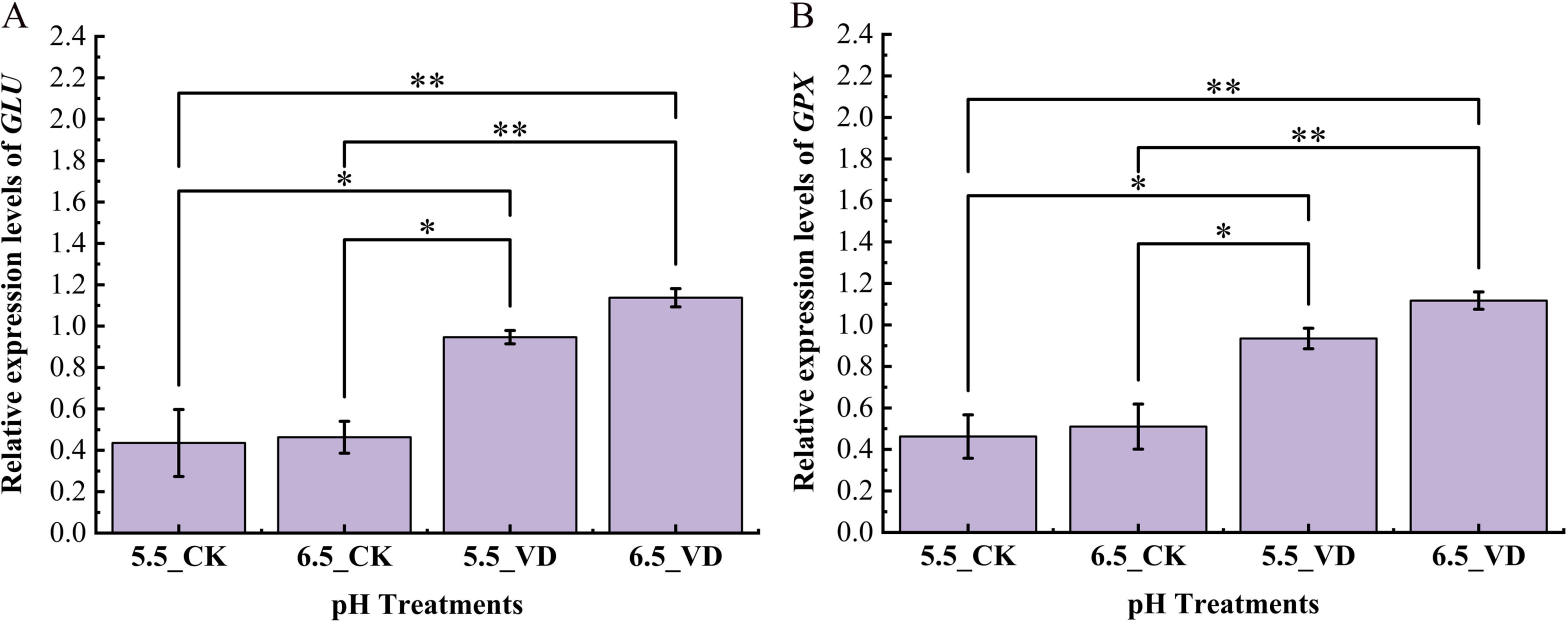
Effect of soil acidification and disease inoculation on *Verticillium* resistant gene expression levels of *SmeGLU* **(A)**, and *SmeGPX* **(B)** in eggplant roots. Error bars indicate the mean ± standard error (n = 4, ***P <0.001, **P < 0.01, *P < 0.05).

The results demonstrated a statistically significant up-regulation of these genes in response to *V. dahliae* infection, particularly under 6.5 pH conditions. Following inoculation, the relative expression levels of *GLU* were significantly higher in plants grown at pH 6.5 (6.5_VD) compared to those at pH 5.5_VD (**Figure 4A**). A similar trend was observed for *GPX* (**Figure 4B**). Specifically, *GPX* expression in 6.5_VD plants was 1.19-fold higher than in 5.5_VD plants. While pH alone did not significantly affect the basal expression of *GLU* and *GPX* in CK plants, it significantly modulated the pathogen-induced expression of these genes. Following *V. dahliae* inoculation, transcript levels of both genes were significantly higher (p< 0.05) at pH 6.5 compared to pH 5.5, indicating that soil pH influences the strength of the eggplant defense gene response.

### 3.4 Effect of soil acidification and *V. dahliae* inoculation on protein localization and concentration of eggplant pathogen resistant genes

**Figure 5:**
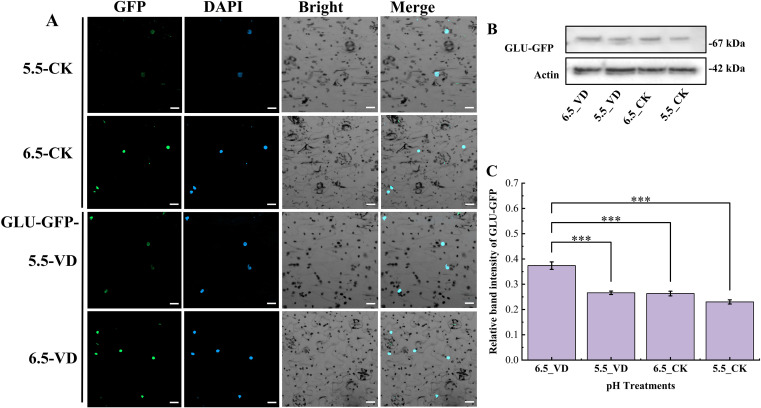
Effect of soil acidification and *V. dahliae* inoculation on protein localization and accumulation in tobacco leaves. GLU-GFP subcellular imaging expressed in agroinfiltrated tobacco plant leaves **(A)**. The GFP signals were visualized using Zeiss confocal microscopy 4 days after infiltration. Bar = 20 µm. Western blot protein bands of GLU-GFP at around 67 kDa with Actin at 42 kDa **(B)**, and Relative bands intensity of GLU-GFP at different pH treated samples **(C)**. The GFP-band intensity was measured using image J and the values were plotted in excel. Error bars indicate the mean ± standard error, with asterisks indicating significant differences (***P <0.001, **P < 0.01, *P < 0.05).

**Figure 6:**
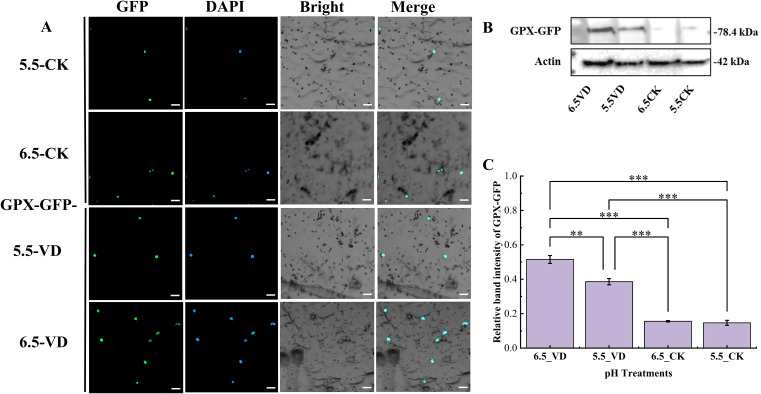
Effect of soil acidification and *V. dahliae* inoculation on protein localization and accumulation in tobacco leaves. GPX-GFP subcellular imaging expressed in agroinfiltrated tobacco plant leaves **(A)**. The GFP signals were visualized using Zeiss confocal microscopy 4 days after infiltration. Bar = 20 µm. Western blot protein bands of GPX-GFP at around 78.48 kDa with Actin at 42 kDa **(B)**, and Relative bands intensity of GPX-GFP at different pH treated samples **(C)**. The GFP-band intensity was measured using image J and the values were plotted in excel. Error bars indicate the mean ± standard error, with asterisks indicating significant differences (***P <0.001, **P < 0.01, *P < 0.05).

According to the subcellular localization, it was observed that the GLU-GFP and GPX-GFP was lower in both control (5.5_CK and 6.5_CK) treatment plants compared to the *V. dahliae* inoculated plants, where higher accumulation of transient protein was localized in cell nucleus (**Figures 5A**, **6A**). According to the immunoblot assays, it was noted that the GLU-GFP release a protein around 67 kDa along with 27 kDa GFP, and 42 kDa Actin (**Figure 5B**). Similarly, the GPX released around 78 kDa protein with 27 kDa GFP (**Figure 6B**). The intensity of the bands varied with the treatment. In both proteins, higher intensity bands were observed in plants at pH 6.5_VD compared to pH 5.5_VD and CK plants (**Figures 5**, **6C**). Quantification revealed that the 6.5_VD treatment resulted in a 1.4-fold and 1.33-fold increase in the expression of GFP-tagged protein for GLU and GPX, respectively, compared to 5.5_VD plants.

### 3.5 Principal component analysis of pH-regulated plant responses to *V. dahliae*

**Figure 7:**
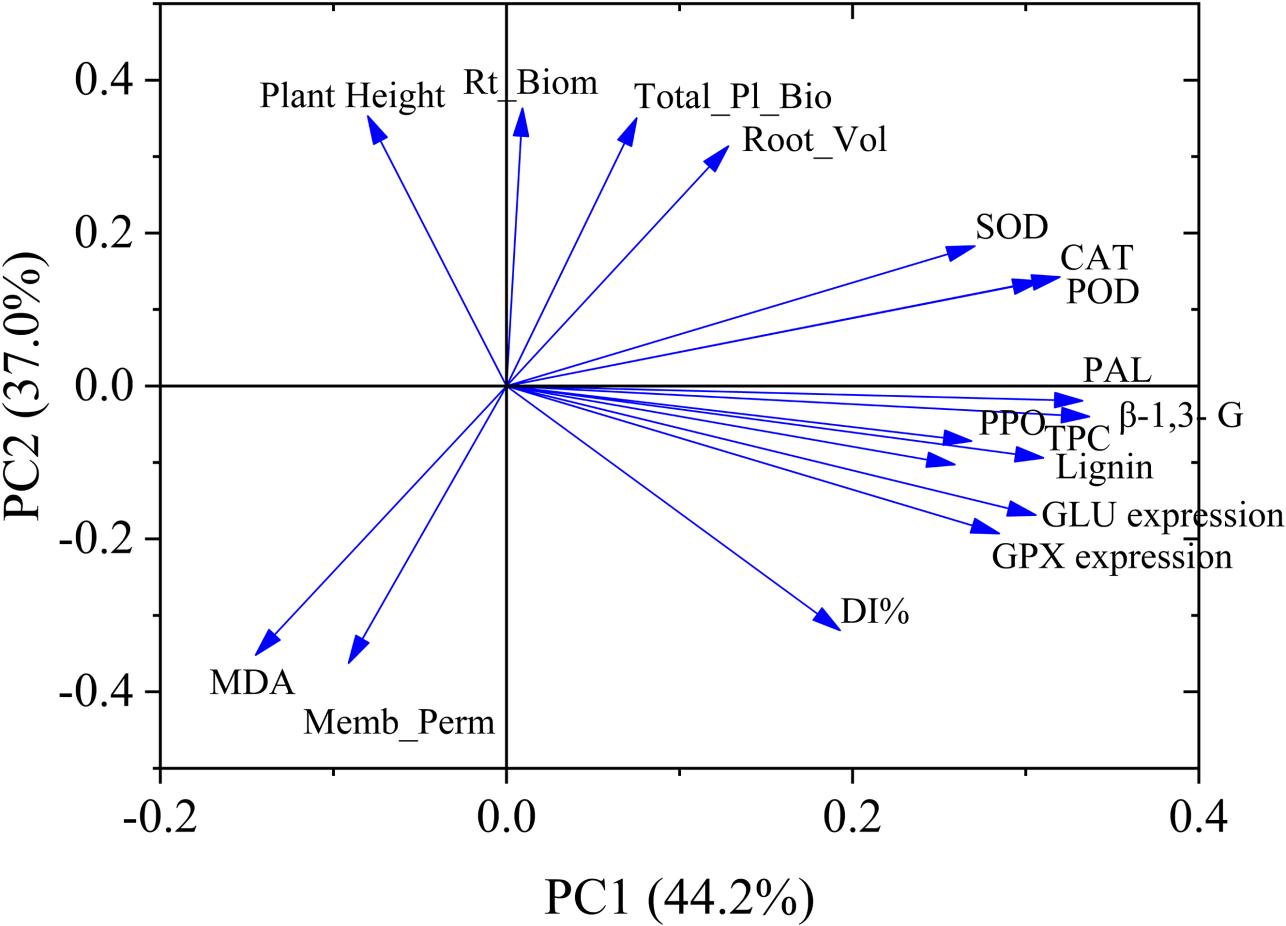
Principal component analysis (PCA) of plant defense parameters under 5.5 and 6.5 pH levels with 95% confidence. Plant height (Pl-Ht), Total plant biomass (Total_Pl_Bio), Root biomass (Rt_Biom), Root volume (R_Vol), SOD, CAT, PAL, β-1, 3-glucanase (β-1, 3-G), PPO, Total phenol content (TPC), Disease index (DI%), Malondialdehyde (MDA), Membrane permeability (Memb_Perm), *GLU* relative genes expression (*GLU* expression), *GPX* relative genes expression (*GPX* expression).

Principal component analysis (PCA) explained 81.2% of the total variance, with PC1 accounting for 44.2% and PC2 for 37.0% of the variation. The PCA biplot clearly separated treatments according to soil pH (5.5 vs. 6.5) and pathogen inoculation status (CK vs. *V. dahliae*), indicating that both factors strongly influenced plant growth, stress physiology, and defense responses (**Figure 7**). Collectively, PCA reveals that soil pH critically modulates the defense-oxidative stress balance during *V. dahliae* infection. A pH of 6.5 favors enzymatic activity, phenolic metabolism, and the expression of defense genes, leading to improved growth and reduced cellular injury. Whereas acidic pH (5.5) exacerbates oxidative stress and membrane damage, resulting in poorer plant performance and higher disease susceptibility.

## 4 Discussion

### 4.1 Effect of soil acidification on defense related enzymatic activity of eggplant

To counteract pathogen attack, plants mobilize sophisticated defense pathways coordinated with robust antioxidant and secondary metabolite systems (Zhou et al., 2012). For Verticillium wilt, plants under optimal conditions typically enhance defense-related enzymatic activities to mitigate damage and bolster resistance (Monazzah et al., 2018). In this study, plants exhibited a significant up-regulation of key defense enzymes (PAL, PPO, β-1, 3-glucanase, POD), antioxidants (CAT, POD, SOD), and metabolites (e.g., lignin, total phenols) at the optimal pH of 6.5 following *V. dahliae* inoculation, compared to suboptimal pH of 5.5_VD/CK. Zhou et al. (2012) reported that the activities of CAT, POD, PPO, and PAL in eggplant were positively correlated with resistance to *V. dahliae*. Suboptimal acidity levels have shown to impair key enzymatic systems compared to optimal conditions.

Peroxidase (POD), a key oxidoreductive enzyme, plays an important role in structural defense by catalyzing processes such as phenol oxidation, suberization, and lignification of cell walls during pathogen response (Jung et al., 2011). Pathogen infection typically elicits increased activity of cell wall-bound peroxidases (Jung et al., 2004). In this study, POD activity was elevated in 6.5-VD pH plants, along with the higher accumulation of lignin and total phenols contents. According to Zhang L. et al. (2015), POD and CAT activities in rice genotypes decreased under lower pH conditions, while similar suppression of SOD and CAT was documented in cucumber plants (Shi et al., 2006). This reduction in enzymatic activity compromises the plant’s capacity to detoxify reactive oxygen species (ROS). Consequently, impaired ROS-scavenging can lead to oxidative damage, including membrane lipid peroxidation and cellular breakdown, thereby increasing susceptibility to pathogen infection (Zhang L. et al., 2015). Infection disrupts cellular osmoregulation, increasing membrane permeability and leading to cytoplasmic exosmosis. This damage, which this study data confirm through elevated relative electrical conductivity (membrane permeability) (**Supplementary Figure 3B**), is further evidenced by a rise in malondialdehyde (MDA) content (**Supplementary Figure 3A**), a direct product and reliable indicator of membrane lipid peroxidation (Radwan et al., 2006). Zhang K. et al. (2015) also documented similar results, showing that acidic conditions triggered an increase in MDA content in the roots of rice. This suggests that suboptimal pH conditions partially constrain the full induction of this crucial structural defense enzyme.

PPO is another defense enzyme that catalyzes the oxidation of polyphenols into antimicrobial quinones. Research indicates that inducing PPO activity can enhance plant resistance to fungal pathogens such as *Fusarium sambucinum* (Ray et al., 1998) and *Fusarium graminearum* (Mohammadi and Kazemi, 2002). It has been reported that the optimum pH for PPO activity varies in different plants. For example, in tea, blueberry, and potatoes, the optimum pH values were 5.0, 6.1, and 7.0, respectively (Jin-long et al., 2009; Siddiq and Dolan, 2017). In the present study, PPO activity was elevated in *V. dahliae*-inoculated 5.5 and 6.5 pH plants compared to CK. This aligns with the findings by Ramamoorthy et al. (2002), who reported increased PPO activity in bacterized tomato roots following challenge with *Fusarium oxysporum*, including the induction of a distinct PPO1 isoform.

The PCA plot confirmed that soil pH is a key driver of plant physiological responses. A pH of 6.5 promoted defense activation and reduced oxidative damage, enhancing resistance. In contrast, acidic pH (5.5) exacerbated oxidative stress and membrane injury, increasing disease susceptibility. Li et al. (2017) reported that genes responsible for disease resistance are significantly inhibited at pH levels below 5.5. This acidic condition favors the pathogenic growth and virulence gene expression (Aro et al., 2005). These finding suggests that the activity of disease resistance genes and enzymes diminishes due to soil acidification.

### 4.2 Effect of soil acidification on key disease resistant gene expression and transient protein expression of eggplant

Rhizosphere pH conditions significantly impair defense genes, weakening plant immunity and increasing disease susceptibility. For example, in *A. thaliana*, the acidic conditions significantly suppressed the important gene (Lager et al., 2010; Lemaître et al., 2008; Singh et al., 2020). In this study, eggplants grown at pH 6.5 exhibited stronger up-regulation of defense-related genes *glutathionine peroxidase* (*SmeGPX*) and *β-1, 3-glucanase* (*SmeGLU*) following *V. dahliae* inoculation. The up-regulation of *SmeGPX* and *SmeGLU* signifies the activation of defense mechanisms against biotic and abiotic stresses (Imran and Ghosh, 2024; Shobade et al., 2025). The translated proteins play critical roles in protecting plants from pathogen attack.

GPXs are crucial antioxidant enzymes that scavenge reactive oxygen species (ROS), converting it to water to mitigate oxidative stress and maintain cellular redox homeostasis (Imran and Ghosh, 2024; Madhu et al., 2023; Passaia and Margis-Pinheiro, 2015). Beyond direct ROS detoxification, GPXs participate in thioredoxin (Trx) and glutathione (GSH) pathways and are implicated in diverse stress and developmental processes (Imran and Ghosh, 2024; Pang et al., 2021). Suboptimal pH conditions in rice downregulated the important genes related to ROS scavenging (Zhang K. et al., 2015). Importantly, Do Carmo Santos et al. (2025) reported that specific GPX isoforms play a role in delaying senescence or reinforcing physical barriers, thereby directly modulating pathogen resistance, with their functions critically linked to cellular localization. This suggests the up-regulated GPX may play a multifaceted role in defense, potentially through both redox signaling and direct protective mechanisms. The up-regulation of SmeGPX in this study aligns with the broader role of the glutathione system in plant defense. Gullner et al. (2018) highlighted that *glutathione S-transferase* (*GST*) genes are strongly induced during biotic stress, and these enzymes work in concert with glutathione peroxidases (GPXs) within coordinated antioxidant network to mitigate pathogen-induced oxidative stress.

*β-1, 3-glucanase (GLU)* gene expression is a marker for resistance in plants against various pathogens (Palacıoğlu, 2024). For example, studies have shown that *Trichoderma harzianum* TIND02 up-regulates PR genes and defense enzymes, including β-1, 3-glucanases, thereby enhancing gray blight resistance in tea (Pandey et al., 2024). At pH levels of 5.5 or below, the expression of disease-resistant genes in tobacco was markedly suppressed, while the expression of pathogenic genes (PopA, PrhA, and SolR) related to bacterial wilt was enhanced. Conversely, the expression of disease-resistant genes was elevated and reached its highest level at pH 6.5 (Li et al., 2017). GLU are pathogenesis-related (PR) proteins belongs to PR2 gene family, they hydrolyze β-1, 3-glucans, the major components of fungal cell walls. By degrading these glucans, β-1, 3-glucanases inhibit fungal growth and development, providing resistance against fungal pathogens (Du et al., 2017; Maksimov et al., 2015; Shobade et al., 2025). This enzymatic activity releases smaller β-1, 3-glucan fragments, which serve as damage-associated molecular patterns (DAMPs) or microbe-associated molecular patterns (MAMPs) (Barghahn et al., 2021; Liu et al., 2023). These oligosaccharide elicitors are recognized by plant pattern recognition receptors (PRRs) on the cell surface, initiating a cascade of immune responses collectively known as pattern-triggered immunity (PTI) (Khan et al., 2025). The activation of PTI leads to transcriptional reprogramming, triggering various defense mechanisms within the plant, including the production of ROS, influx of Ca²^+^ ions, and activation of mitogen-activated protein kinase (MAPK) cascades (Li et al., 2024b; Nabi et al., 2024).

In wheat, β-1, 3-glucanase and POD functions synergistically improve cell wall lignification and reduce callose deposition during infestation by *Diuraphis noxia*, effectively strengthening the cell wall and protecting the plant (Zondo and Mafa, 2025). Similarly, *Bacillus* species can induce defense mechanisms and elicit the expression of PR-protein genes, including β-1, 3-glucanases, in tomato against *Rhizoctonia solani* (Zahoor et al., 2025). *Ve*-mediated resistance in tomato, involves lignin accumulation, which restricts MS survival even in crop residues.

The upregulation of SmeGLU, and SmeGPX highlight their roles in combating *V. dahliae*. Both of these transient proteins were expressed and localized in the cell nucleus with higher accumulation was recorded in plants at optimum pH 6.5 with disease inoculation compared to CK plants in both pH, suggests that optimal pH supports post-transcriptional stability and translation efficiency. Conversely, acidic pH may promote protein degradation or inhibit translational machinery, weakening defense responses and up-regulate the virulence genes of *V. dahliae*. These findings underscore the importance of soil pH management in controlling Verticillium wilt, as even minor shifts can tip the balance in favors of the pathogen. Moreover, the correlation analysis revealing a significant relationship between plant disease resistance, pathogen severity and soil pH. Under optimal pH environments, plants exhibit stronger growth, heightened disease resistance, and optimum production of pathogenesis-related proteins (SmeGLU and SmeGPX) and these are the conditions that are unfavourable for pathogen development.

## 5 Conclusions

This study provides the first evidence that soil acidification critically determines eggplant resistance to *V. dahliae* under greenhouse conditions. A near-neutral pH of 6.5 created an optimal physiological state, enhancing disease resistance through the coordinated up-regulation of key defense enzymes (PAL, PPO, β-1, 3-glucanase, POD), antioxidants (CAT, POD, SOD), phenolic metabolites (lignin, total phenols), and the defense-related genes *SmeGLU* and *SmeGPX* and translated proteins. These findings reveal soil pH management as a viable strategy for disease mitigation. This study provides comprehensive physiological, biochemical, transcriptional, and protein-level evidence associating soil pH to eggplant resistance against *V. dahliae*, but it does not include functional validation of candidate defense genes. Future research should functionally validate the roles of *SmeGLU* and *SmeGPX*, for example through heterologous overexpression and VIGS to elucidate their specific contributions to pH-modulated defense.

## Data Availability

The original contributions presented in the study are included in the article/**Supplementary Material**. Further inquiries can be directed to the corresponding authors.
