# Supplementary material for: pH-dependent defense responses in eggplant: growth performance and resistance to Verticillium dahliae
Source: Front Plant Sci. 2026 Mar 12;17:1785225. doi: 10.3389/fpls.2026.1785225 (PMC13019319; doi:10.3389/fpls.2026.1785225)
Supplement: Supplementary file 1 [file DataSheet1.docx]

Supplementary Material

# Supplementary Table S1 The initial, middle and final soil pH of each treatment during the experiment.

| **pH** | **5.5_CK** | **6.5_CK** | **5.5_VD** | **6.5_VD** |
| --- | --- | --- | --- | --- |
| **Initial** | 5.44±0.03 | 6.45±0.03 | 5.44±0.03 | 6.45±0.03 |
| **Middle** | 5.56±0014 | 6.46±0.08 | 5.59±0.13 | 6.45±0.13 |
| **Final pH** | 5.73±0.05 | 6.62±0.08 | 5.73±0.01 | 6.48±0.1 |

**Supplementary Table S2**. List of primers used for gene expression levels

| **Gene** | **Gene ID** | **Forward Primer (5'-3')** | **Reverse Primer (5'-3')** | **TM** | **GC%** |
| --- | --- | --- | --- | --- | --- |
| IAA27 | KY859268 | TTGCCTGGTTCTGAATCACC | CCCATCTCCCAGAATCACCA | 57.8/59.8 | 50/55 |
| MPK1 | KY861322 | TGAGGAGCACTGCCAGTATT | CGAGCTAGCCCAAAATCACA | 57.8/57.8 | 50/50 |
| GPX | Smechr0502112.1 | AGTGCTGTTGGAAATCGAGT | AGCCAGCATTAACAAGTTCGA | 55.7/56 | 45/42.8 |
| CHT | Smechr1200301.1 | AGAGAACAAGGTAGCCCAGG | AATGGGGCCTCTTCCGAAAT | 59.8/57.8 | 55/50 |
| GLU | Smechr0102781.1 | GGATCAAACATCGAAGTCATGC | ACCCACCAATTCGCATGTTC | 58.2/57.8 | 45.45/50 |
| PR1 | Smechr0100501.1 | AAGAATTGGAGACTGCCGGA | TGCTTCTCATCGACCCACAT | 57.8/57.8 | 50/50 |
| PR5 | Sme2.5_30700.1 | CAAACACCCTGGCTGAATACG | ACTAGGATTGGTCGGTGCAA | 59.97/57.8 | 52.38/50 |
| LOX | AB244527.1 | AGCGCTTTGGTCACTTGAAG | GGGCATGTAATTCAGGGAGG | 57.8/59.8 | 50/55 |
| Actin | JX524155.1 | CTCCCACATGCCATTCTTCG | GATTTCTCGCTCAGCTGTGG | 59.8/59.8 | 55/55 |

**Supplementary Table S4 List of GFP tag primer sequences used for downstream applications**

| **Gene** | **Primer F/R (5’-3’)** | **TM** |
| --- | --- | --- |
| **GPX-1300-BAMHI-F** | GAGCTCGGTACCCGGGGATCCATGGCCGGCCAACCGGAGAAG | 65 |
| **GPX-1300-SALI-R** | GCCCTTGCTCACCATGTCGACCTCGATTCCCAGCAGCACTTTTATGTCCCTC |  |
| **GLU-1300-BAMHI-F** | GAGCTCGGTACCCGGGGATCCATGGCTACCTCACAAATGGTTG | 58 |
| **GLU-1300-SALI-R** | GCCCTTGCTCACCATGTCGACCATCTCACTAATGAGAGAGACAGTAGC |  |

The restriction enzyme cutting sites are indicated in red

**Supplementary Figure S1:**

#
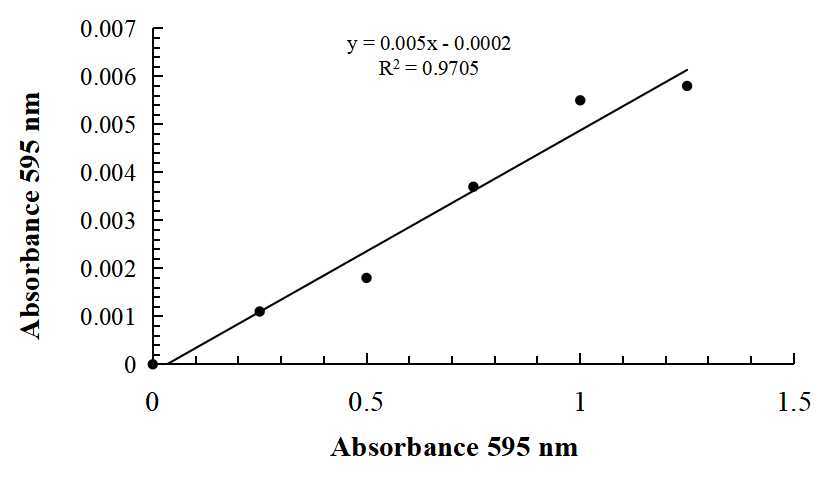


**Figure S1**. A representative Bradford standard assay curve of concentrations vs. absorbance. BSA (Bovine serum albumin) was used as standard and diluted to the 0, 0.25, 0.5, 0.75, 1.0, and 0.125 mg/ml concentrations, and absorbance was scored at 595 nm wavelength.

**Supplementary Figure S2**


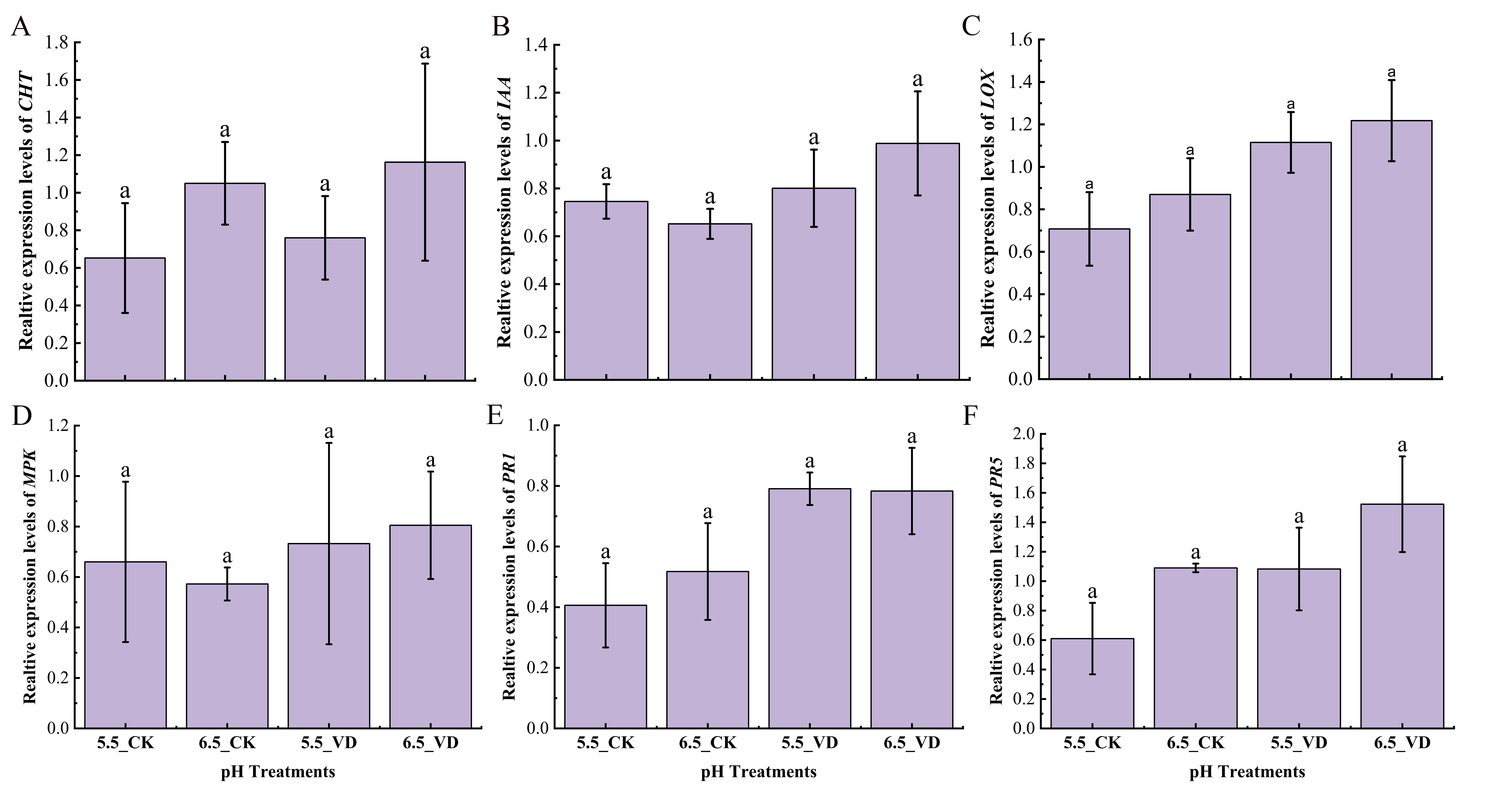


**Figure S2**. Effect of soil acidification and *Verticillium* inoculation on disease resistant gene expression levels of *SmeCHT* (A), *SmeIAA* (B), *SmeLOX* (C)*, SmeKPK1* (D)*, SmePR1* (E)*,* and *SmePR5* (F) in eggplant roots. Error bars indicate the mean ±standard error (n = 4, NS).

**Supplementary Figure S3**


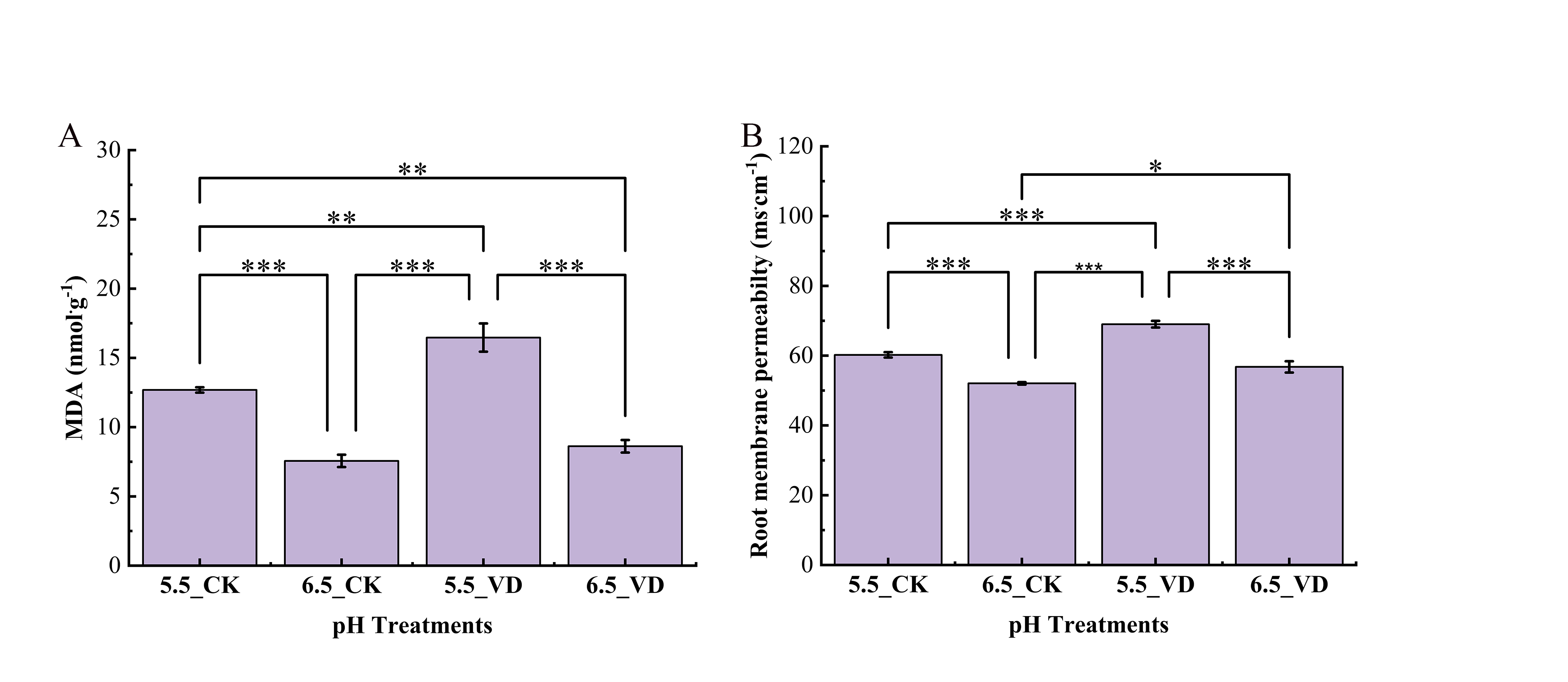


**Figure S3**. Effect of soil acidification and disease inoculation on (A) malondialdehyde (MDA) content and (B) root membrane permeability of eggplant roots. Error bars indicate the mean ±standard error (n = 4, ****P* ＜0.001, ***P* ＜ 0.01, **P* ＜ 0.05).
